# Polyelectrolyte-Functionalized Nanofiber Mats Control the Collection and Inactivation of *Escherichia coli*

**DOI:** 10.3390/ma9040297

**Published:** 2016-04-19

**Authors:** Katrina A. Rieger, Michael Porter, Jessica D. Schiffman

**Affiliations:** Department of Chemical Engineering, University of Massachusetts Amherst, Amherst, MA 01003-9303, USA; krieger@umass.edu (K.A.R.); koizumi011@gmail.com (M.P.)

**Keywords:** antibacterial, bacteria, cellulose, chitosan, electrospun, inactivation, nanofiber, poly (acrylic acid), polyelectrolyte

## Abstract

Quantifying the effect that nanofiber mat chemistry and hydrophilicity have on microorganism collection and inactivation is critical in biomedical applications. In this study, the collection and inactivation of *Escherichia coli* K12 was examined using cellulose nanofiber mats that were surface-functionalized using three polyelectrolytes: poly (acrylic acid) (PAA), chitosan (CS), and polydiallyldimethylammonium chloride (pDADMAC). The polyelectrolyte functionalized nanofiber mats retained the cylindrical morphology and average fiber diameter (~0.84 µm) of the underlying cellulose nanofibers. X-ray photoelectron spectroscopy (XPS) and contact angle measurements confirmed the presence of polycations or polyanions on the surface of the nanofiber mats. Both the control cellulose and pDADMAC-functionalized nanofiber mats exhibited a high collection of *E. coli* K12, which suggests that mat hydrophilicity may play a larger role than surface charge on cell collection. While the minimum concentration of polycations needed to inhibit *E. coli* K12 was 800 µg/mL for both CS and pDADMAC, once immobilized, pDADMAC-functionalized nanofiber mats exhibited a higher inactivation of *E. coli* K12, (~97%). Here, we demonstrate that the collection and inactivation of microorganisms by electrospun cellulose nanofiber mats can be tailored through a facile polyelectrolyte functionalization process.

## 1. Introduction

Electrospinning is an established and versatile technique that enables the production of mats composed of non-woven nano-/micro-scale diameter fibers [1,2,3,4]. The fabricated fibers are continuous and have high axial strength and extreme flexibility. Due to the interconnectivity of the fibers, the assembled nanofiber mats have microscale interstitial spaces, large surface-to-volume ratios, and incredibly high porosity. Additionally, specific functions can be incorporated into electrospun mats to broaden their application from passive textiles to multifunctional materials for water and air purification, biocatalysis supports, and biosensors [5,6,7,8].

Notably, electrospun mats also hold great promise when tailored towards biomedical applications, such as scaffolds for the treatment of chronic wounds and tissue engineering, because nanofibers promote hemostasis, fluid absorption, cell respiration, and gas permeation [7]. The physical and chemical properties of electrospun nanofiber mats can be readily modified by encapsulating and/or immobilizing bioactive species to elicit specific biological responses. For example, it has been reported that fiber diameter [9], alignment [10,11], porosity [12], and surface functionalization [10,11,13] all significantly affect the ability of mammalian cells to adhere and proliferate. When considering the interface between nanofiber mats and microbiology, the majority of literature has focused on developing antibacterial mats [7], by encapsulating antibiotics [14,15] or functional nanoparticles [16] into the nanofiber mats.

However, detailed, quantitative interfacial studies between microorganisms and electrospun nanofiber mats are limited. Previously, Abrigo *et al.* [17,18] qualitatively reported that the average diameter of polystyrene fibers influenced the ability of microbes to proliferate and colonize within the nanofiber mats. They qualitatively explored the influence of fiber surface chemistry on microbial behavior by plasma polymerizing four monomers (acrylic acid, allylamine, 1,7-octadiene, and 1,8-cineole) onto the polystyrene fibers [18]. Additionally, we have recently quantified the microbial uptake capacity of cellulose nanofiber mats using three medically relevant and distinct microorganisms, Gram-negative *Escherichia coli* K12 and *Pseudomonas aeruginosa* PA01, as well as the Gram-positive *Staphylococcus aureus* MW2 [19]. By pairing experimental data with a quasi-equilibrium model and diffusion kinetics of *E. coli* K12 removal, we provided insight into the properties and parameters that result in high microorganism removal using cellulose nanofiber mats. These limited reports demonstrate that fiber diameter and surface chemistry can be strategically tuned to control microbial behavior; however, further fundamental studies that quantify the interactions between nanofiber mats and microorganisms are needed.

In this study, we aim to quantify the collection and inactivation of *E. coli* K12 as a function of nanofiber mat surface charge and hydrophilicity. This work focuses on physically adsorbing an ultra-thin layer of cationic or anionic polymer onto a “green” polysaccharide template; specifically, cellulose nanofibers hydrolyzed from electrospun cellulose acetate nanofibers. Cellulose was chosen because it is the most abundant natural polymer, hydrophilic, has negative surface charges, is insoluble in water, and is a common material used for biocompatible products that interface with microorganisms [20,21,22,23]. In this study, we successfully functionalized the cellulose nanofiber mats using poly (acrylic acid) (PAA, a weak polyanion), chitosan (CS, a weak polycation), and polydiallyldimethylammonium chloride (pDADMAC, a strong polycation). Surface functionalization was conducted via a facile coating process, which enables endless surface chemical variety and retention of the original underlying morphology [24]. Here, the most hydrophilic nanofiber mats (cellulose and pDADMAC-functionalized) provided an equally high collection of *E. coli* K12, whereas the presence of ~NH_2_^+^ and/or ~NH^+^ groups from the polycation-functionalized nanofiber mats (CS- and pDADMAC-functionalized) provided antimicrobial properties [25,26,27] during viability studies.

## 2. Materials and Methods

### 2.1. Materials and Chemicals

Low molecular weight chitosan (LMW CS, poly (d-glucosamine), Mw = 460,000 Da), low molecular weight polydiallyldimethylammonium chloride (LMW pDADMAC, 20 wt % in water, Mw = 100–200 Da), poly (acrylic acid) (PAA, 35 wt % in water, Mw = 250,000 Da), glycerol (1,2,3-propanetriol glycerin, ≥99%), propium iodide solution (PI, 1.0 mg/mL in water), diiodomethane (≥99.0%), Mueller Hinton broth (MHB), and ReagentPlus^®^ grade acetic acid (AA, ≥99.0%) were obtained from Sigma-Aldrich (St. Louis, MO, USA). SYTO^®^ 9 green fluorescent nucleic acid stain (S34854, 5 mM solution in dimethyl sulfoxide) was purchased from Life Technologies (Grand Island, NY, USA). Sodium hydroxide (NaOH) was purchased from Fisher Scientific (Fair Lawn, NJ, USA). Difco Luria-Bertani (LB) broth was purchased from BD Life Sciences (Franklin Lakes, NJ, USA). Deionized (DI) water was obtained from a Barnstead Nanopure Infinity water purification system (Thermo Fisher Scientific, Waltham, MA, USA).

### 2.2. Cellulose Nanofiber Mat Fabrication

A 15 w/v% solution of cellulose acetate in acetone [28] was mixed for 24 h at 20 rpm using an Arma-Rotator A-1 (Elmeco Engineering, Bethesda, MA, USA). The solution was loaded into a 5-mL Luer-Lock tip syringe capped with a Precision Glide 18 gauge needle (Becton, Dickinson & Co., Franklin Lakes, NJ, USA), which was secured to a PHD ULTRA™ syringe pump (Harvard Apparatus, Plymouth Meeting, PA, USA). Alligator clips were used to connect the positive anode of a high-voltage supply (Gamma High Voltage Research Inc., Ormond Beach, FL, USA) to the needle and the negative anode to a copper plate wrapped in aluminum foil. A constant feed rate of 3 mL/h, an applied voltage of 25 kV, and a separation distance of 10 cm were used to spin cellulose acetate nanofiber mats. The assembled electrospinning apparatus was housed in an environmental chamber (CleaTech, Santa Ana, CA, USA) with a desiccant unit (Drierite, Xenia, OH, USA) to maintain a temperature of 22 °C ± 1 °C and a relative humidity of 55%. In this study, all cellulose acetate nanofiber mats were electrospun for 1 h before being converted to cellulose nanofiber mats. As-spun mats sandwiched between Teflon sheets were thermally treated at 208 °C for 1 h and then submerged in a 4/1 v/v% solution of H_2_O/ethanol containing 0.1 M NaOH for 24 h [29,30]. The mats were then washed using DI water and placed in a desiccator for 24 h at room temperature (23 °C) to dry.

### 2.3. Functionalization of Electrospun Cellulose Mats with Polyelectrolytes

A CS solution was prepared by first dissolving CS in 0.5 M AA at 2.5% w/v ratio and then diluting to a 0.5% w/v CS solution in 0.5 M AA. PAA and pDADMAC solutions were prepared by diluting the as-received stock solution to 0.5 w/v% in DI water. Before use, each diluted solution was first vortexed using a Fisher Scientific Analog Vortex Mixer (02215365). To coat, first an electrospun mat was punched into a circle with a 2.54 cm diameter using a Spearhead^®^ 130 Power Punch MAXiset (Fluid Sealing Services, Wausau, WI, USA). Six mats at a time were submerged in a square petri dish containing 20 mL of one of the coating solutions described above. The petri dish was then placed onto a 120 V Lab Line 3-D rotator (model #4630, Thermo Scientific, Waltham, MA, USA) for 30 min. After rotation, the mats were removed from solution, rinsed with DI water, and dried for 24 h.

### 2.4. Characterization of Polyelectrolye-Functionalized Electrospun Mats

Static contact angles were measured using an in-house apparatus equipped with a Nikon camera. Each fiber mat was adhered to a glass microscope slide to ensure that the sample was flat when the picture was taken. To form a droplet on the fiber mat sample, a solution (~10 µL) of glycerol was dropped from above the sample using a glass pipet. DI water and diiodomethane were also used, but the droplets immediately absorbed into the samples. Data was collected in triplicate and analyzed using Image J 1.45 software (National Institutes of Health, Bethesda, MD, USA).

Micrographs were acquired using a FEI-Magellan 400 scanning electron microscope (SEM, FEI, Hillsboro, OR, USA). A sputter machine (Gatan high-resolution ion beam coater model 681, Cressigton Scientific Instruments, Watford, UK) was used to coat samples with ~5 nm of platinum. Fiber diameter distribution was determined using Image J 1.45 software (National Institutes of Health, Bethesda, MD, USA) by measuring 50 random fibers from 5 micrographs. A Fourier transform infrared spectrometer (FTIR, PerkinElmer Spectrum 100, Waltham, MA, USA) confirmed the regeneration of cellulose after alkaline treatment of the as-spun cellulose acetate nanofiber mats. Additional spectra was acquired on cellulose nanofiber mats functionalized with PAA, CS, and pDADMAC, along with PAA, CS, and pDADMAC bulk controls. PAA and pDADMAC were cast into films and CS was tested as a powder.

A Zeiss Axiovert 4-laser spinning disc confocal microscope (Zeiss confocal, ×20 magnification, Zeiss, Jenna, Germany) was used to collect z-stack composite images of cellulose nanofiber mats fluorescently stained with calcofluor white stain (1 μL/mL). The 3D composite images from Zen software (Zen lite LSM 800, Zeiss, Jenna, Germany) were imported into Image J 1.45 software from which the average thickness of the nanofiber mats was determined by averaging 50 thickness measurements taken from 5 different nanofiber mats. The total internal surface area of the nanofiber mats was estimated using an Autosorb^®^-iQ system (Quantachrome, Boynton Beach, FL, USA) using 50 mg of the electrospun nanofiber mat that were degassed for 2 h at 150 °C. The total surface area was calculated for the nanofiber mat using the Brunauer-Emmett-Teller (BET) method [31].

High-resolution scans were obtained using X-ray photoelectron spectroscopy (XPS, Physical Electronics Quantum 2000 Microprobe, Physical Electronics Inc., Chanhassen, MN, USA) to determine the chemical composition using the known sensitivity factors. A monochromatic Al X-rays at 50 W was used with a spot area of 200 µm, and the take-off angle was set to 45°.

### 2.5. Quantification of Bacteria Uptake by Polyelectrolye-Functionalized Electrospun Mats

*Escherichia coli* K12 (*E. coli* K12) was purchased from Leibniz Institute DSMZ (Braunschweig, Germany). Bacteria were grown in LB at 37 °C and re-suspended in a phosphate buffered saline solution (PBS, pH 7.2) to remove residual macromolecules and other growth medium constituents. Throughout the experiment, no external forces were applied.

Using 6-well plates, an electrospun mat punched into a circle with a diameter of 2.54 cm was incubated in a bacterial solution with an initial concentration of 1.52 × 10^8^ cells/mL (5 mL per well). A control sample (no mat, bacteria solution only) was run in parallel to each experiment, and six trials for each type of mat were performed. For all experiments, the mats were incubated for 2 h at 37 °C at 150 rpm, over which a portion of the bacteria transferred from the surrounding solution to the mat. The optical density of both the sample and control well were monitored using the McFarland 0.5 standard, which is equal to 1–2 × 10^8^ cells/mL [32]. Concentrations were measured using an absorbance microplate reader (BioTek ELx800™, BioTek Instruments Inc., Winooski, VT, USA) at an absorbance of 600 nm. A calibration curve was developed to convert the microplate readings to optical densities, and then to cell concentrations. These concentrations were confirmed using plate counting. To calculate the total number of cells removed by the mat after 120 min, we calculated the difference between the concentration of bacteria in the sample well containing a mat and the concentration of bacteria in the control well (no mat).

### 2.6. Evaluation of Antibacterial Activity of Polyelectrolyte-Functionalized Electrospun Mats

Minimum inhibitory concentration (MIC) was determined for CS and pDADMAC based on a previously outlined procedure [32]. An overnight culture of *E. coli* K12 was prepared in MHB. A Fisherbrand polypropylene 96-well plate was filled with an increasing concentration gradient of the CS and pDADMAC coating solutions, along with a Gentamycin antibiotic control. The concentrations of the CS and pDADMAC solutions started at 12.5 µg/mL and doubled at each well until 6400 µg/mL. The Gentamycin control started from 0.03 µg/mL and doubled until 16 µg/mL. Two columns of the well plate remained controls: The growth control contained MHB and bacteria and the sterile control contained only MHB. After the well plate incubated (37 °C) for 20 h, the bacteria concentrations in each well were measured using an absorbance microplate reader at an absorbance of 600 nm.

Viability loss was determined using a previously described fluorescence assay [33]. Electrospun mats (2.54 cm diameter) were individually placed in a 6-well plate (Becton, Dickinson & Co., Franklin Lakes, NJ, USA). Cells (10^7^ cells/mL) resuspended in an isotonic solution were diluted by a factor of 2 and added to each well in 5-mL portions. The cells were incubated at 37 °C for 180 min. After 180 min, cells were stained in the dark with PI (excitation/emission at 535 nm/617 nm) for 15 min and then counter-stained with SYTO^®^ 9 stain (excitation/emission at 358 nm/461 nm). Fluorescence images were acquired utilizing an epifluorescence microscope (Zeiss) with a Chroma cube filter. Five representative images were taken at ×20 magnification at various locations for each specimen. Dead cells and the total number of cells were determined by direct cell counting. The percentage of dead cells (or loss of viability) was determined from the ratio of the number of cells stained with PI divided by the number of cells stained with SYTO^®^ 9 plus PI. Throughout the Results and Discussion section, all statistical differences were determined using an unpaired *t*-test with values of *p* ≤ 0.05 considered to be statistically significant.

## 3. Results and Discussion

### 3.1. Characteristics of Polyelectrolyte-Functionalized Cellulose Nanofiber Mats

**Figure 1:**
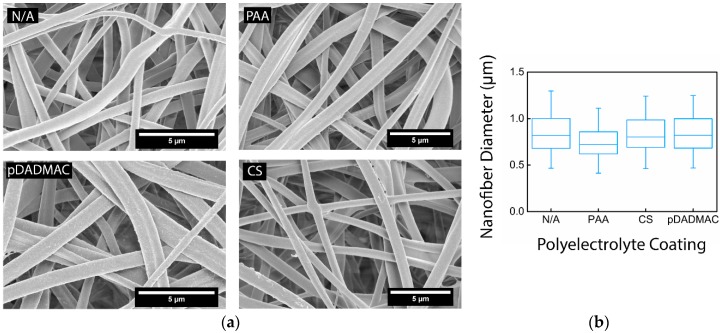
(**a**) SEM micrographs of electrospun cellulose nanofiber mats without functionalization (N/A) and functionalized with PAA, CS, and pDADMAC. All scale bars are 5 µm; (**b**) A box-and-whisker plot shows the median, lower and upper quartile, and the minimum and maximum value for the nanofiber diameter distribution for the cellulose nanofiber mats without functionalization (N/A) and functionalized with PAA, CS, and pDADMAC. All nanofiber mats had a statistically equivalent average fiber diameter. *N* = 3 nanofiber mats to confirm nanofiber morphology. Fiber diameter distribution was determined by measuring 50 random fibers from 5 micrographs.

**Figure 2:**
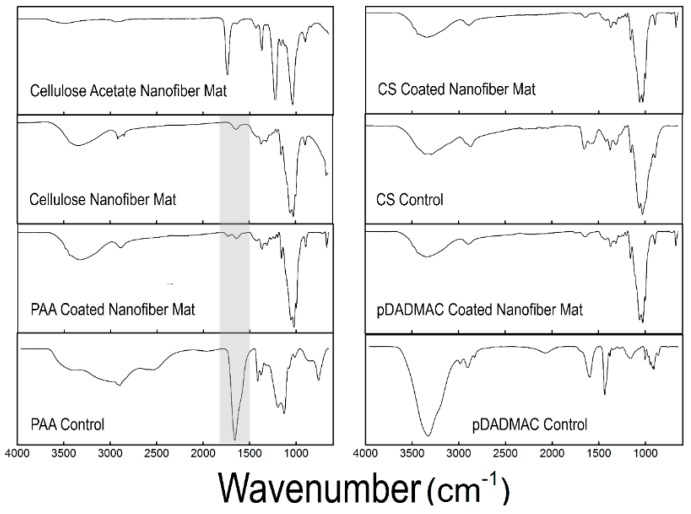
FTIR spectra of the as-spun cellulose acetate nanofiber mat and the regenerated cellulose nanofiber mat are displayed, along with the spectra for cellulose nanofiber mats functionalized with PAA, CS, and pDADMAC. Control spectra for PAA, CS, and pDADMAC are also provided. The highlighted region shows the addition of a peak in the 1700 cm^−1^ region that correlates with C=O of carboxylic acid for the PAA-functionalized cellulose nanofiber mats. FTIR spectra was acquired on *N* = 3 samples.

For this study, cellulose nanofiber mats were chosen to serve as the basis for polyelectrolyte functionalization. Cellulose acetate solutions were electrospun for 1 h before being regenerated into nanofiber mats comprised of cellulose (Figure 1). Fourier transform infrared spectra acquired on the as-spun cellulose acetate and the cellulose nanofiber mats confirmed that cellulose acetate had been regenerated to cellulose (Figure 2) [19]. Specifically, the disappearance of the 1750 cm^−1^ peak indicated that the acetate groups had been replaced with hydroxyl groups [29]. The resulting regenerated cellulose nanofibers were smooth, with a slight ribbon morphology. The average diameter of the cellulose nanofibers was determined to be 0.85 ± 0.22 µm by analyzing micrographs acquired using scanning electron microscopy (SEM) (Table 1). Using confocal microscopy, it was determined that the nanofiber mats electrospun for this work had a bulk thickness of 42.4 ± 12 μm. The total surface area of the cellulose nanofiber mats was estimated using the Brunauer-Emmett-Teller (BET) method to be 4.5 m^2^/g. In general, the regenerated cellulose nanofiber mats were consistent with regard to their fiber diameter [28,29,30], morphology [30,34], and surface area [35] to cellulose nanofiber mats previously reported in the literature.

Cellulose nanofiber mats were functionalized with one of three different polyelectrolytes: poly (acrylic acid) (PAA), an anionic polymer, as well as chitosan (CS) and polydiallyldimethylammonium chloride (pDADMAC), which are cationic polymers. The average fiber diameter for each sample after undergoing the coating process was determined; there was no statistical change in average fiber diameter (Table 1). Post-functionalization with PAA and CS, the nanofiber surface appeared smooth and the fiber morphology stayed intact (Figure 1). Nanofiber mats functionalized with pDADMAC appeared to have a textured surface, but there was no indication of aggregation or that the surface functionalization was uneven.

Elemental data acquired using XPS for carbon, nitrogen, and oxygen are summarized in Table 1. Nanofiber mats functionalized with CS and pDADMAC showed a statistical increase in nitrogen *versus* the control cellulose nanofiber mats. Functionalization using either pDADMAC or CS should result in the adsorption of positively charged amine groups to cellulose’s negatively charged hydroxyl groups due to electrostatic interactions [36,37]. Thus, this statistical difference supports the hypothesis that surface functionalization with polycations was achieved. Consistent with previous literature [38], there was no statistical difference in the elemental data acquired on the control cellulose nanofiber mats and those functionalized with PAA.

**Figure 3:**
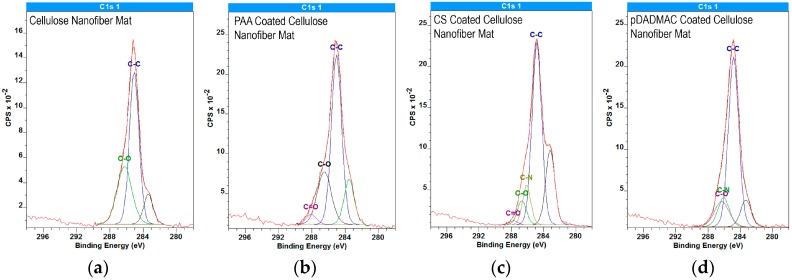
High-resolution C1s XPS spectra for (**a**) cellulose nanofiber mats; (**b**) PAA; (**c**) CS; and (**d**) pDADMAC-functionalized cellulose nanofiber mats. The individual contributions to the data from different functional groups such as C-C (285.0 eV), C-O (286.6 eV), and C-N (286.1 eV), and C=O (287.9 eV) are provided. The unmarked curve at 283 eV is likely due to the morphologically heterogeneous nature of the nanofiber mats. XPS data was acquired on *N* = 2 nanofiber mats, which were scanned at three unique locations per sample.

Figure 3 highlights the presence of PAA on the PAA-functionalized cellulose nanofiber mats; the PAA-functionalized C1s spectrum show the presence of C=O component at 287.9 eV, which is absent in the cellulose C1s spectra. C1s spectra for cellulose and PAA-functionalized cellulose nanofiber mats both resolve into contributions centered at 285.0 eV from the C-C and C-H functionalities. Additionally, at 286.6 eV, the C-O contribution of hydroxyl groups is present in both spectra. The unmarked curve at 283 eV is likely caused by “loose” nanofibers, which have different neutralization time scales than bulk material, thus leading to morphologically heterogeneous samples.

Further chemical analysis was performed using FTIR. A comparison of control cellulose nanofiber mats (no functionalization) with mats functionalized with PAA, CS, and pDADMAC, as well as, bulk PAA, CS, and pDADMAC controls is shown in Figure 2. No significant changes can be seen in the FTIR spectra after functionalization using CS or pDADMAC. However, an additional peak in the 1700 cm^−1^ region that correlates with the C=O of carboxylic acid became present after the cellulose nanofiber mats were functionalized with PAA [39]. The presence of this second peak (Figure 2, highlighted region) confirms that PAA functionalization on the cellulose nanofiber mats was successful.

**Figure 4:**
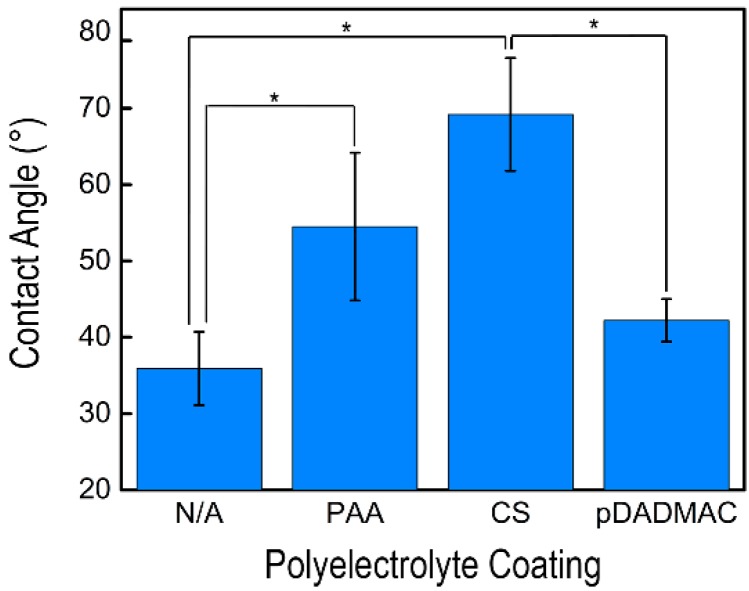
The average glycerol contact angle on cellulose nanofiber mats without functionalization (N/A) and functionalized with PAA, CS, and pDADMAC are shown. Error bars represent standard deviation, and the statistical significance determined using an unpaired t-test between samples is shown by an *. Contact angle measurements were acquired on *N* = 5 samples.

Contact angle measurements of the cellulose nanofiber mats with and without polyelectrolyte functionalization are shown in Figure 4 and on Table 1; measurements were performed using three solutions: water, glycerol, and diiodomethane. Both water and diiodomethane immediately absorbed into all nanofiber mats with or without a polyelectrolyte functionalization, thus prohibiting the acquisition of a measurement. Cellulose nanofiber mats had a glycerol contact angle of 35.9° ± 4.8°, which is consistent with the low contact angle reported by others [40]. The contact angle measurements acquired on cellulose nanofiber mats functionalized with PAA were statistically increased over the cellulose nanofiber mats [41,42]. Functionalization with CS resulted in the highest glycerol contact angle of 69.2° ± 7.4° [43]; a statistically higher contact angle was acquired on samples functionalized with CS *versus* non-functionalized and pDADMAC-functionalized cellulose nanofiber mats. Statistically speaking, the PAA- and CS-functionalized samples had the same contact angle. However, it should be noted that a high standard deviation of contact angles was acquired for both the PAA- and CS-functionalized nanofiber mats. The high standard deviation could insinuate that less functionalization occurred by these polyelectrolytes than by the pDADMAC. There was no statistical difference in contact angles between control cellulose nanofiber mats and the cellulose nanofiber mats functionalized with pDADMAC. Overall, all of the nanofiber mats, both non-functionalized and functionalized, were hydrophilic as the contact angles were all <90°.

Overall, functionalization of cellulose nanofiber mats with different polyelectrolytes provides an effective method for adding chemical groups onto the material’s surface while keeping the morphology, fiber diameter, and high surface area constant. Additionally, electrospinning polyelectrolytes usually involves co-spinning with a synthetic polymer, harsh solvents, or a post-crosslinking. This simple post-functionalization technique was facile and effective, and avoided these issues.

### 3.2. Collection of E. coli K12 by Polyelectrolyte-Functionalized Cellulose Nanofiber Mats

**Figure 5:**
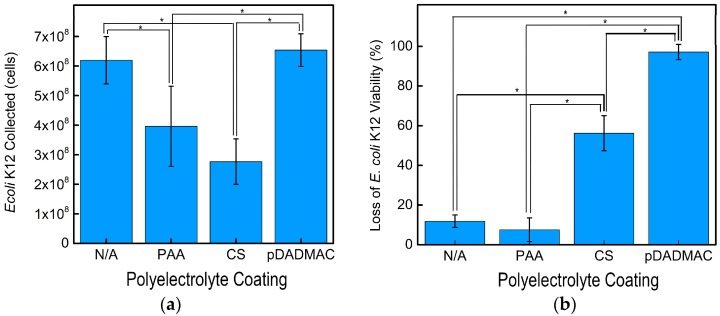
(**a**) The average number of *E. coli* K12 collected after 120 min using cellulose nanofiber mats without functionalization (N/A) and functionalized with PAA, CS, and pDADMAC; (**b**) The average loss of viability for *E. coli* K12 after 180 min using cellulose nanofiber mats without functionalization (N/A) and functionalized with PAA, CS, and pDADMAC. Collection and viability data reported for *N* = 3 samples. Error bars represent standard deviation and the statistical significance determined using an unpaired t-test between samples is shown by an *.

Cellulose nanofiber mats with and without polyelectrolyte functionalization were incubated with *E. coli* K12 for 120 min to demonstrate the effect that surface charge has on microbial collection (Figure 5a). The highest *E. coli* K12 collection was achieved by the control electrospun cellulose nanofiber mats and those functionalized with pDADMAC. After 120 min, the highly hydrophilic cellulose and pDADMAC-functionalized cellulose nanofiber mats (Figure 4) removed a statistically equivalent amount of *E. coli* K12, 6.2 × 10^8^ ± 8.0 × 10^7^ and 6.5 × 10^8^ ± 5.5 × 10^7^ cells, respectively. Despite also having cationic charge groups, nanofiber mats functionalized with CS collected the lowest number of cells (2.8 × 10^8^ ± 7.7 × 10^7^ cells), which equated to a statistically lower removal than the pDADMAC-functionalized and control cellulose nanofiber mats. The surface of both Gram-positive and Gram-negative bacterial cells have net negative charges. This suggests that, by enhancing electrostatic interactions via increasing the concentration of positive charges on a materials’ surface, that potentially, a higher bacterial adsorption, could be achieved [44]. While both pDADMAC and CS have positive charges, adhesion has also been reported to depend on other interactions, such as hydrophobic/-philic interactions [44]; based on the contact angle measurements, pDADMAC-functionalized nanofiber mats are more hydrophilic. Additionally, *E. coli* K12 contains lipopolysaccharides (LPS) on their cell envelope, thus attracting a water layer that leads to a high level of hydrophilicity [44], which is likely to enhance cell adhesion to hydrophilic surfaces.

The nanofiber mats functionalized with PAA had a statistically lower amount of *E. coli* K12 cells collected, 4.0 × 10^8^ ± 1.4 × 10^7^ cells, than the control (non-functionalized) or pDADMAC-functionalized cellulose nanofiber mats. The PAA-functionalized nanofiber mats did collect more *E. coli* K12 than the CS-functionalized nanofiber mats likely due to their lower contact angle and negative surface charge. Previous studies have reported similar results. A higher collection of bacteria was achieved by hydrophilic metals that had a positive or neutral surface charge compared to hydrophilic negatively charged substrates [45]. When taken collectively, the statistical changes in nanofiber mat contact angle and total number of bacterial cells collected support that a simple polyelectrolye functionalization on cellulose nanofiber mats can provide a tailored collection of microorganisms.

### 3.3. Inactivation of E. coli K12 by Polyelectrolyte-Functionalized Cellulose Nanofiber Mats

In addition to altering the microbial collection rate, the presence of cationic charges should provide innate microbial inactivation. We conducted a solution-based minimum inhibitory concentration (MIC) evaluation using *E. coli* K12 for both CS and pDADMAC polymers. Both polymer solutions had an MIC value of 800 µg/mL, which was similar to those reported in the literature [25,46]. MICs are known to vary based on molecular weight. Previously, CS MICs for *E. coli*, *P. aeruginosa*, and *S. aureus* have been reported to range between 100–1000, 200–1700, and 20–1250 µg/mL, respectively [46]. The MIC of pDADMAC was absent from the literature but some values have been reported for polymers containing DADMAC monomers [27]. Notably, the antibacterial activity of the polymer in solution does not necessarily correlate to its activity once the polymer is immobilized on a solid material [47] because the mechanism by which the antibacterial agent and the microorganisms interact changes, thus changing the stresses induced on the microorganisms.

The inactivation of *E. coli* K12 by the nanofiber mats was measured after 180 min of incubation (Figure 5b). The cellulose nanofiber mats (non-functionalized) and the nanofiber mats functionalized with PAA, exhibited a minimal loss of *E. coli* K12 viability, 11.8% ± 3% and 7.5% ± 6%, respectively. This was expected because these polymers do not contain cationic groups to provide antibacterial activity. In applications such as microbial fuel cells, cellulose, and PAA-functionalized nanofiber mats could provide a platform for tailored biofilm growth. Both mats provide high cell viability and the carboxyl groups of PAA could be used to further functionalize the nanofiber mat.

Both CS and pDADMAC are cationic polymers and have intrinsic antibacterial activity; thus, we expected these two functionalizations to lead to antimicrobial nanofiber mats. Functionalizing the cellulose nanofiber mats with CS statistically increased the inactivation activity to 56.3% ± 9%. Our previous report indicated that chitosan-based nanofiber mats inactivated >99% *E. coli* K12; thus, a higher inactivation might be achieved if the chitosan content on the surface of the nanofibers was increased [47]. The statistically highest inactivation (97.2% ± 4%) was achieved by the electrospun nanofiber mats functionalized with pDADMAC. While the MIC experiment indicated that both CS and pDADMAC have the same solution-based antibacterial activity against *E. coli* K12, the higher level of inactivation demonstrated by the pDADMAC-functionalized nanofiber mats suggests that, when immobilized, pDADMAC has a higher inactivation potential than CS. Another hypothesis is that a higher degree of functionalization occurred using pDADMAC than CS and that the overall quantity of cationic polymer on the surface of the cellulose nanofiber mats is different. Previously, the electrostatic interactions between pDADMAC and cellulose nanofiber mats has been used to “seed” the surface of the mats with zeolites. SEM micrographs confirmed that a uniform decoration of zeolites on the surface of the fibers was only enabled by a consistent pDADMAC layer [37]. Additionally, previous literature has demonstrated that a minimal amount of chitosan was absorbed onto cellulose films, but a greater functionalization could be achieved after the oxidation of cellulose because there were more anionic sites available [36]. This suggests that further processing of the cellulose nanofiber mats could increase CS functionalization if desired.

## 4. Conclusions

In this study, the influence of nanofiber mat surface chemistry and hydrophilicity on microbial interactions was investigated by functionalizing the surface of cellulose nanofiber mats with PAA, CS, and pDADMAC. We have demonstrated that, while the polyelectrolyte functionalization did not change the fiber morphology or their average fiber diameter, the surface chemistry, and hydrophilicity of the fiber mats were impacted. Hydrophilicity paired with neutral or positive charge improved the collection of *E. coli* K12, whereas hydrophilic cationic nanofiber mats exhibited the highest killing of *E. coli* K12. We suggest that insights gained from this work could enable the fine-tuning of high porosity nanofiber mats towards a desired end application. By optimizing mat hydrophilicity and surface chemistry, a balance of microorganism collection *versus* repulsion, as well as microbial viability *versus* killing, can be achieved.

## Figures and Tables

**Table 1:** Summary of the materials properties of polyelectrolyte-functionalized electrospun cellulose nanofiber mats.

Polyelectrolyte Coating	Average Fiber Diameter (μm)	Contact Angle (°) ^1^	XPS ^2^ (Atomic %)		
			C	N	O
N/A	0.85 ± 0.22	35.9 ± 4.8	56.2 ± 4.3	-	41.6 ± 1.8
PAA	0.75 ± 0.20	54.5 ± 9.7	56.3 ± 5.6	-	42.5 ± 4.6
CS	0.84 ± 0.21	69.2 ± 7.4	62.5 ± 3.3	2.3 ± 1.4	35.2 ± 4.0
pDADMAC	0.89 ± 0.32	42.2 ± 2.8	57.8 ± 1.6	1.3 ± 0.9	40.9 ± 1.8

^1^ Glycerol contact angle is reported. The water and diiodomethane contact angles were also tested, but the solutions absorbed immediately into all nanofiber mats. Contact angle measurements were acquired on *N* = 5 samples; ^2^ XPS: X-ray photoelectron spectroscopy.

## Abbreviations

The following abbreviations are used in this manuscript:

BET	Brunauer-Emmett-Teller
CS	Chitosan
*E. coli* K12	*Escherichia coli* K12
FTIR	Fourier transform infrared spectroscopy
PAA	Poly(acrylic acid)
pDADMAC	Polydiallyldimethylammonium chloride
SEM	Scanning electron microscopy
XPS	X-ray photoelectron spectroscopy
